# Community-Based Telehealth Approach Improves Specialist Access for Individuals with Increased Cancer Risk in Low-Resource Settings

**DOI:** 10.3390/cancers17081317

**Published:** 2025-04-14

**Authors:** Aksel Alp, Winston Doud, Christian Doud, Thair Takesh, Cherie Wink, Annachristine Miranda-Hoover, Joseph Foote, Rongguang Liang, Diana V. Messadi, Anh Le, Petra Wilder-Smith

**Affiliations:** 1 Beckman Laser Institute and Medical Clinic, School of Medicine, University of California Irvine, Irvine, CA 92612, USA; aksela@uci.edu (A.A.); wdoud@uci.edu (W.D.); cdoud@ucmerced.edu (C.D.); ttakesh@uci.edu (T.T.); cwink@hs.uci.edu (C.W.); 2 Department of Oral and Maxillofacial Surgery/Pharmacology, School of Dental Medicine, University of Pennsylvania, Philadelphia, PA 19104, USA; ahoover@upenn.edu (A.M.-H.); joseph.foote@pennmedicine.upenn.edu (J.F.); anh.le@pennmedicine.upenn.edu (A.L.); 3 Department of Oral & Maxillofacial Surgery, Perelman Center for Advanced Medicine, Penn Medicine Hospital of the University of Pennsylvania, Philadelphia, PA 19104, USA; 4 Wyant College of Optical Sciences, University of Arizona, Tucson, AZ 85719, USA; rliang@optics.arizona.edu; 5 Section of Oral Medicine, UCLA School of Dentistry, Los Angeles, CA 90095, USA; dmessadi@dentistry.ucla.edu

**Keywords:** oral cancer, telehealth, low-resource settings, specialist, compliance

## Abstract

**Background/Objectives**: The low-resource, minority and underserved populations (LRMU) that carry the highest risk of oral cancer (OC) experience many barriers to early detection and treatment, resulting in disproportionately poor outcomes. One major barrier to better outcomes is poor compliance with specialist referral for diagnosis and treatment. The goal of this prospective study was to compare specialist referral compliance for Telehealth vs. in-person visits in LRMU individuals screening positive for increased OC risk. **Methods**: Forty subjects who had screened positive for oral potentially malignant lesions (OPMLs) were recruited from community clinics. The subjects indicated whether they would prefer an in-person or Telehealth specialist visit. They were offered assistance with all aspects of the visit, and then tracked over 3 months for referral compliance. A novel, very low-cost, simple Telehealth platform located within the community clinic was used for the remote specialist visits. **Results**: In the Telehealth group, 16/24 subjects attended their first scheduled remote specialist visit; 4/24 attended rescheduled visits within 3 months, and 4/24 did not comply at all. All attendees and specialists were able to complete the remote visits in full. Of the 7/16 subjects who completed in-person visits, 3/16 attended their first scheduled visit, and 4/16 complied within 3 months; 9/16 subjects did not comply at all with specialist referral. Significantly more individuals complied with Telehealth specialist referral at 1 month (*p* = 0.0006) and after 3 months (*p* = 0.0154). **Conclusions**: This novel Telehealth platform may improve compliance with specialist referral in low-resource individuals with OPMLs.

## Simple Summary

Individuals in low-resource settings carry a disproportionately high risk of oral cancer, yet they lack access to specialist diagnosis and care, leading to poor outcomes. While inadequate screening accuracy presents one barrier to better outcomes, another major hurdle is poor specialist referral compliance. The goal of this prospective study was to evaluate the impact of a novel Telehealth platform on specialist referral compliance. The prototype Telehealth system is tailored for use in non-specialist community settings. Of the 40 subjects with increased oral cancer risk who were enrolled in this study, 24 opted for Telehealth, and 20/24 completed a remote specialist visit within 3 months. Sixteen selected an in-person specialist visit, and seven of the sixteen attended their in-person visit within 3 months. Significantly more individuals complied with Telehealth specialist referral at 1 month (*p* = 0.0006) and after 3 months (*p* = 0.0154). Based on these results, this tailored community-based Telehealth platform may improve specialist access in individuals from low-resource settings undergoing specialist referral for increased oral cancer risk.

## 1. Introduction

Oral and oropharyngeal cancer (OC) is the sixth most common cancer globally, and the only major cancer whose outcomes continue to worsen. While this situation is in part related to the rising global prevalence of HPV, the primary barriers to better outcomes remain unchanged: late diagnosis and delayed treatment [1,2,3,4,5]. While OC treatment outcomes are good when the condition is diagnosed at an early stage, the morbidity and mortality are high for later-stage lesions [6,7,8,9].

The majority of oral cancers are presumed to develop from oral potentially malignant lesions (OPMLs) [10]. These lesions typically can be detected by visual inspection during screening. However, differentiating OPMLs from benign lesions can be challenging, and existing tools are unable to predict malignant changes in premalignant lesions, which require regular monitoring to ensure the early detection of any progression towards malignancy. The typical pathway to care for an individual with an oral lesion presents unusual challenges that all constitute barriers to early diagnosis and treatment. Persons from low-resource, minority and underserved (LRMU) populations carry the highest OC risk [6,7,8,9,11]. Unfortunately, these populations have limited access to regular dental and medical care, such that they are not routinely screened for OC risk [11]. Dentists and hygienists are mandated to screen routinely for OC risk at every visit. This screening step is essential, because it is a positive risk-screening outcome that triggers specialist referral for surgical biopsy, diagnosis and either treatment or heightened monitoring. Even when individuals from LRMU populations have access to screening, their compliance with specialist referral is constrained by challenges that include cost, fear, unfamiliarity with specialist and academic centers, family care duties, lack of transportation, language barriers and cultural barriers [12,13]. Thus, specialist referral compliance from these individuals is typically low for all branches of medicine, ranging from 18 to 55% [14,15,16,17,18].

A recent study in an LRMU population of individuals who had screened positive for increased OC risk reported that 83% of the individuals complied with a Telehealth OC specialist referral within 6 months, whereas only 30% of subjects complied with an in-person specialist referral. Of the individuals who attended the first specialist appointment, approx. 85% then attended a second follow-up in-person specialist visit, so that after 6 months, 72.5% of the Telehealth subjects had entered into the continuum of care vs. 25% of the individuals who had opted for the initial in-person specialist visit [19].

Studies in other areas of medicine have confirmed the value of Telehealth in overcoming barriers to care. This field of research was greatly expanded during the COVID-19 epidemic [20,21,22,23,24,25,26,27]. For example, in one pediatric study in a low-resource setting (LRS), investigators found that in-person specialist referral compliance increased by a factor of three after a preceding remote visit with the specialist [28]. While Telehealth has the potential to overcome many of the greatest barriers to improving OC outcomes, its implementation in the field of oral health remains limited. One extensive systematic review reported that, even when Telehealth is employed in the OC realm, an asynchronous communication mode is typically used. Unfortunately, this indirect form of communication forfeits most of the benefits of Telehealth [29].

Our overall hypothesis is that providing a remote option for the first specialist visit can overcome many of the critical barriers to compliance. The objective of this study was to evaluate the effectiveness of a remote intraoral camera-based Telehealth platform prototype in improving access to OC specialists in LRMU patients suspected of OPML. The specific aims included the following: (a) comparing patient compliance with in-person vs. Telehealth specialist referral, and (b) determining the efficacy and ease of use of remote specialist intraoral examination. The long-term goal is to improve specialist referral compliance for LRMU individuals with increased OC risk in order to ensure better and more equitable health outcomes.

## 2. Materials and Methods

This project was conducted in full compliance with the University of California, Irvine’s IRB-approved protocol #2002-2805. Written informed consent was obtained from all subjects involved in the study, all of whom completed the study in accordance with the approved protocols.

### 2.1. Subjects

Forty subjects who had screened positive for OPMLs at Concorde College of Dental Hygiene in Garden Grove, CA, West Coast University Dental Hygiene Clinic in Anaheim, CA, and the University of California, Irvine’s clinics were recruited. The standard of care screening process used by all dentists and hygienists, which includes visual examination, palpation and risk factor assessment, was used (https://www.aaom.com/clinical-practice-statement--oral-cancer-screening (accessed on 12 November 2024)). The subject demographics are shown in Table 1.

### 2.2. Protocol

After completing the in-person standard OC risk screening, patients with a positive screening outcome for OPMLs were provided with full details about this study and asked whether they would like to participate. These individuals were told that non-participation would not affect their treatment in any way. Those who opted into the study provided written informed consent. Using identical language each time, subjects were asked to indicate whether they preferred a conventional referral to an in-person specialist or a Telehealth in-home visit with a specialist. The subjects were told that neither form of specialist visit would incur any costs for them. The individuals who chose the Telehealth option were given a return appointment at the community clinic for a remote specialist visit that included the services of an interpreter as needed. The individuals who selected the in-person specialist visit were asked to select a specialist from the list of providers to the clinics participating in this study. The subjects were offered whatever assistance they needed at no cost to them, including family care, transportation, an interpreter to accompany them on their specialist visit and the ability to bring one support person, such as a family member or friend, with them to the appointment.

### 2.3. Telehealth Specialist Visit

The Telehealth visit was completed in the community clinic. Staff noted the travel time to and from the clinic for each subject, as well as the instruction and Telehealth visit durations. They also recorded any assistance that the subject received with regard to transportation, family care and interpreter services. Next, the research staff briefly showed the subject how to operate the Telehealth system, and after that timepoint, they only assisted the subject if absolutely necessary. Researchers recorded how many such interventions were necessary and the reasons for them. During the remote specialist inspection, the patient moved the intraoral camera wand around the oral cavity according to instructions over a smartphone from the remote oral medicine specialist. The remote visit continued until a full 8-point visual examination of the mouth had been completed. Patients who were non-compliant with the first scheduled remote specialist visit received a weekly text message or phone call reminder encouraging them to reschedule. Communications and compliance were recorded throughout the study.

### 2.4. Telehealth System

**Figure 1:**
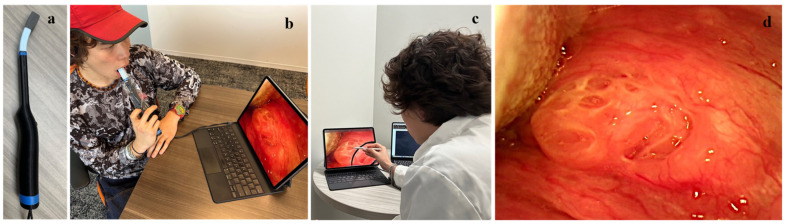
(**a**) Imaging tip of Telehealth system has 90° of flexibility to ensure easy, gag-free imaging access throughout oral cavity, including tonsillar pillars and sublingual areas. (**b**) Telehealth system during use by patient. (**c**) Synchronous specialist view during remote examination. (**d**) High-resolution image from Telehealth probe seen simultaneously in real time by patient and specialist.

The HIPAA-compliant prototype Telehealth system utilized in this study consists of a prototype intraoral camera, a computer tablet with Wi-Fi connection and a bridging application (app). The app consists of two components: (1) an app installed on the study tablet that allows a patient to view their oral cavity on the screen display; and (2) a partner app installed on the remote specialist’s computer that automatically interfaces with Zoom, allowing for the secure synchronous viewing of the patient’s oral cavity, as well as the ability to capture still and video images during use. Figure 1 shows the prototype Telehealth system and its usage. The intraoral camera has a unique 180-degree flexible neck, enabling it to be adjusted for optimal viewing of all areas of the mouth and the tonsils. It also features autofocus functionality with a long focal range, allowing for imaging access to the entire oral cavity, including the tonsillar pillars, floor of the mouth, posterior buccal region and all palatal regions, while minimizing contact with the oral tissues to avoid triggering the gag reflex.

The prototype Telehealth system (Figure 1a) was designed to integrate seamlessly with the Zoom Telehealth app for ease of use for both the patient and the remote specialist. For each Telehealth session, the remote specialist initiated contact with the patient using Zoom. This triggered a notification on the study tablet instructing the patient to accept the call, thus establishing the connection between the intraoral camera, study tablet and screen sharing with the specialist. Prior to each use of the intraoral camera, a disposable sterile sheath was applied. During each remote visit, the specialist provided verbal instructions to the patient regarding the probe placement and angulation as they completed a full intraoral visual examination (Figure 1b). The specialist was able to capture still images and/or record video footage throughout the call, similar to any standard Zoom Telehealth call (Figure 1c,d). After the remote session, the patient rated the Telehealth platform’s ease of use and their satisfaction with the Telehealth experience on a visual analog scale (VAS) ranging from 0 to 10. The subjects were also asked to indicate their preference between Telehealth appointments and in-person consultations for future appointments (Yes/No).

### 2.5. Statistical Analysis

The study data were analyzed using the GraphPad Prism 10.4.2. software (GraphPad Software, Inc. Boston, MA, USA). A Fisher exact test was used to compare the proportions of the subjects who showed compliance, with *p* < 0.05 set as the level of significance.

## 3. Results

Twenty-four subjects opted for a Telehealth specialist visit, and sixteen chose to attend an in-person specialist appointment. The demographics of the two study groups were comparable (Table 1). Approximately two-thirds of the subjects in each group were non-native English speakers. Interestingly, 67% of females opted for Telehealth visits vs. 56% of males, and the mean age of the remote specialist group was 5 years older than that of the in-person visit group.

Of the 24 subjects who requested a Telehealth visit, 7/24 requested an interpreter, 24/24 said they would bring a family member for support, 2/24 requested assistance with family care and 8/24 requested transportation to and from the community clinic. Sixteen were non-native English speakers. The 20/24 subjects who actually attended their Telehealth visit spent an average of 26 min each way traveling to the community clinic, 4 min learning how to use the Telehealth system and 28 min on the Telehealth call. Their mean score for the Telehealth ease of use was 8.1/10, and their mean overall satisfaction with the Telehealth visit scored 8.7/10 (Table 2), which was significantly better (*p* = 0.0116) than the 6.9/10 patient satisfaction with the in-person visit. All subjects in the Telehealth group were able to operate the intraoral camera and the Zoom Telehealth App satisfactorily. The specialists were also able to complete full intraoral inspections of all patients. Two individuals required some help from the clinic assistant to initiate the Zoom meeting.

Ten of the sixteen individuals who requested an in-person specialist visit were non-native English speakers. Six of them requested an interpreter, all sixteen indicated that they would bring a family member to the office visit, two requested assistance with family care and eleven needed transportation to the in-person appointment. The travel time to the specialist’s office averaged 67 min each way, and on average, the office visit lasted 26 min. The subjects’ mean overall satisfaction with the in-person visit scored 6.9/10 (Table 2).

In the Telehealth group, 16/24 subjects attended the first scheduled remote specialist visit; 4/24 attended rescheduled visits within 3 months, and 4/24 did not comply at all. Of the 7/16 subjects who completed the in-person visit, 3/16 attended the first scheduled visit, and 4/16 complied within 3 months; 9/16 did not comply at all with specialist referral (Table 3).

67.5% of the subjects complied with any form of specialist referral within 3 months. The initial compliance (attending the first scheduled visit) was significantly better in the Telehealth group: 66.7% vs. 18.6%. The three-month referral compliance was also significantly better in the Telehealth group: 83.3% vs. 43.6%. (Table 3).

## 4. Discussion

The premise of this study is that, if specialist access for LRMU individuals with increased OC risk can be improved, healthoutcomes will also benefit. The fact that OC outcomes are especially poor in this high-risk group with little specialist access adds further urgency to finding a scalable solution to this challenge.

The first step in the pathway to OC management is non-specialist screening for OC risk, typically by dentists or hygienists who are mandated to complete a risk assessment during office visits. Unfortunately, conventional screening is only 40–60% accurate [30,31]. Various technologies and approaches are being developed and evaluated to overcome this first barrier in the pathway to care, some with very promising results. However, improved screening and triage will only translate into better OC outcomes if the patients who screen positive have a workable pathway to specialist access and utilize it. Because most OC specialists are situated in academic or hospital centers in large cities, accessing them can be arduous, costly and daunting, especially for individuals who are not familiar with such settings.

During the COVID-19 pandemic, Telehealth emerged as an important modality for providing healthcare to patients [20,21,22,23,24,25,26,27]. Mandate-compliant communication, record-keeping and reimbursement pathways were also developed and validated. It is on this foundation that the current study is based, with the goal of identifying a viable pathway to bridge the gap from screening to specialist diagnosis and subsequent management in LRMU individuals with increased OC risk.

In this study, participants were asked to select the specialist visit option of their choice, rather than being randomized to the in-person or remote options, in order to identify which form of specialist visit the patients preferred, and to maximize the potential for referral compliance. Interestingly, older patients did not prefer the in-person option to a greater extent than the younger study participants. The mean age of the Telehealth group was 5 years greater than that of the in-person group. The four oldest subjects in this study all opted for a remote specialist visit. Thus, pre-study concerns that permitting the patients to choose their specialist visit mode might result in a much younger population in the Telehealth group were unfounded. These results are in agreement with the findings of some other studies that also reported a slight preference for Telehealth amongst older individuals [32,33]. Perhaps older individuals are more reluctant to leave their homes and travel long distances, especially when most of them use cellphones on a daily basis [34]. In this study, the travel time to the in-person specialist appointment was more than twice as long as to the Telehealth appointment, which may well have served as a deterrent to completion of the in-person visit. Other studies have reported that barriers in patient confidence [35,36,37], as well as a low ability to use telemedicine, lack of literacy, lack of assistance from others and physical and cognitive disabilities, may contribute to the reduced acceptance of Telehealth modalities by the elderly [35,36,37]. In this study, the design of the prototype Telehealth technology and its usage were specifically tailored to overcome some of these potential hurdles; by situating the Telehealth visit in a local and familiar community location, the subjects were less anxious. They could be shown how to use the technology by the staff and helped as needed. Moreover, the patients were encouraged to bring a friend or family member to the Telehealth visit to assist as needed and to reduce their anxiety, and a translator was readily available to assist with any language barriers.

Most study participants were not native English speakers. Because a very similar percentage of each group (66.7% vs. 62.5%) fell into this category, the potential effect of this variable on patient conduct was considered minimal.

Twice as many individuals from low-resource populations selected the Telehealth option vs. the in-person specialist visit, despite all of the potential and perceived barriers to remote care. This finding supports the premise that Telehealth can be suitable for implementation in low-resource populations, independent of age or other demographics. Similar findings were reported for many areas of Telehealth. Thus, nine million people alone used telemedicine services under Medicare after the onset of the COVID-19 crisis [38]. Early data showed its ready adoption across all races and ethnicities [39]. Nearly USD 4 billion was billed nationally for non-Medicare Telehealth visits during just two months of 2020—March and April—compared to less than USD 60 million for the same two months of 2019 [39].

Overall, referral compliance varied significantly between the two modes of specialist visit. Only 18.6% of subjects attended the first in-person specialist visit, and 43.6% of the individuals in this group complied with referral within 3 months. In the remote appointment group, 66.7% of the patients attended their first Telehealth visit, and 83.3% completed their Telehealth specialist visit within 3 months. The considerable difference in compliance between the two groups is similar to that reported from an earlier study in another low-resource population [19]. Furthermore, in that study, approximately 85% of individuals who complied with either form of first visit subsequently complied with a second in-person specialist visit for entry into the pathway of care. That finding underlines the importance of tailoring the first specialist visit to the individual needs of the patient who is undergoing specialist referral, as it appears that a successful first specialist visit provides the basis for excellent compliance with in-person follow-up appointments and entry into the pathway of care. Oral cancer outcomes are primarily determined by the cancer stage at the time of diagnosis, and an increase in the time up to treatment of as little as 2 months significantly increases the risk of death [4,40]. Therefore, earlier and better compliance with specialist referral by individuals in low-resource settings can help to address the inequitably poor oral cancer outcomes in this population [41,42,43,44].

While this study was carefully designed to achieve its stated goals, it had several weaknesses. The study population was limited in size in this pilot study. As a consequence, some of the sample sizes in the individual response groups were quite small, which may limit their generalizability. Selection bias may have affected the data, as these were all individuals attending free dental clinics who had agreed to participate in this study. Less adventurous individuals may have ruled themselves out of participation. Because the subjects were permitted to choose their desired form of specialist visit, the group sizes were not comparable, and there may have been bias in the subjects’ compliance and behavior. The decision was made to allow the subjects to follow their preference because this approach parallels current practice in many areas of medicine. Patients are now routinely offered a choice between in-person and Telehealth visits in many spheres of medical care, especially since the COVID-19 pandemic. Moreover, the Telehealth protocol followed in this study during the remote specialist visits parallels the process as we envisage it in future use in community settings.

Another issue that merits further investigation is the role that the regular reminder communications and the provision of child/elder care, transportation and interpreters may have played in promoting referral compliance. Moreover, further research, especially prospective and long-term studies evaluating the effects of Telehealth technologies on health outcomes, is needed to understand the long-term impact of telemedicine on disease progression and patient outcomes, especially in the light of the cultural and linguistic differences which may affect communication and medical decision-making in telemedicine. Moreover, potential approaches to overcoming some of the unique challenges posed by telemedicine need to be evaluated, such as specialized training, facilities and equipment.

Overall, the study hypothesis that providing a remote option for the first specialist visit can overcome many of the critical barriers to compliance was confirmed. Larger randomized studies that address these variables are now under way to further explore these questions.

## 5. Conclusions

The implementation of a novel, community-based Telehealth platform significantly improved specialist referral compliance for oral cancer risk in individuals from low-resource settings. Larger studies in different settings and patient demographics are needed to solidify these findings.

## Figures and Tables

**Table 1:** Study population demographics.

	Remote Specialist Visit	In-Person Specialist Visit
Mean Age (Years)	61	56
Age Range (Years)	32–76	38–69
Median Age (Years)	58	53
Asian	6	3
Pacific Islander	0	1
White Non-Hispanic	5	4
White Hispanic	9	6
African American	2	1
Mixed Race	2	1
Female	10	5
Male	14	11
Total	24	16

**Table 2:** Comparison of logistics: Telehealth vs. in-person specialist visit groups.

	Remote Specialist	In-Person Specialist	Total
	n (%)	n (%)	n (%)
Requested Interpreter	7/24 (29.2%)	6/16 (37.5%)	13/40 (32.5%)
Non-Native English Speaker	16/24 (66.7%)	10/16 (62.5%)	26/40 (65%)
Brought Family Members	24/24 (100%)	16/16 (100%)	40/40 (100%)
Needed Transportation	8/24 (33.3%)	11/16 (68.75%)	19/40 (47.5%)
Help with Family Care	2/24 (8.3%)	2/16 (12.5%)	4/40 (10%)
Mean Travel Time to Specialist Appointment	26 min	67 min	
Mean Specialist Visit Duration (plus Telehealth Training)	32 min	26 min	

**Table 3:** Compliance with specialist visit over time.

	Remote Specialist	In-Person Specialist	Remote vs. In-Person Comparison (Sig: *p*-Value)		Total
	n (%)	n (%)	*p*-Value	*p*-Value Summary	
Compliance with Specialist Referral @ First Appointment	16/24 (66.7%)	3/16 (18.6%)	0.0006	**Sig.**	19/40 (47.5%)
Compliance with Specialist Referral within 3 Months, but After Missed First Appointment	4/24 (16.7%)	4/16 (25%)	0.6905	N.S.	8/40 (20%)
Total Compliance with Specialist Referral within 3 Months	20/24 (83.3%)	7/16 (43.6%)	0.0154	**Sig.**	27/40 (67.5%)
Mean Patient Satisfaction (Out of 10) with Specialist Visit	8.7/10(87%)	6.9/10(69%)	0.0116	**Sig.**	N.A.

## Data Availability

Data supporting the reported results may be available upon request.
